# Imaging-Driven Risk Stratification and Endovascular Decision Pathways in Acute Pulmonary Embolism

**DOI:** 10.3390/diagnostics16081200

**Published:** 2026-04-17

**Authors:** Fabio Corvino, Francesco Giurazza, Massimo Galia, Antonio Corvino, Pierleone Lucatelli, Antonio Basile, Marcello Andrea Tipaldi, Cristina Mosconi, Raffaella Niola

**Affiliations:** 1 Interventional Radiology Department, AORN “A. Cardarelli”, 80131 Naples, Italy; francesco.giurazza@aocardarelli.it (F.G.);; 2 Section of Radiology, Department of Biomedicine, Neuroscience and Advanced Diagnostics (BiND), University Hospital “Paolo Giaccone”, 90127 Palermo, Italy; 3 Medical, Movement and Wellbeing Sciences Department, University of Naples “Parthenope”, 80133 Naples, Italy; 4 Vascular and Interventional Radiology Unit, Department of Radiological, Oncological and Pathological Sciences, Sapienza University of Rome, Policlinico Umberto I, Viale del Policlinico 155, 00161 Rome, Italy; 5 Department of Medical Sciences, Surgical and Advanced Technologies, University Hospital Policlinic “G. Rodolico-San Marco”, 95125 Catania, Italy; 6 Interventional Radiology Unit, Department of Surgical and Medical Sciences and Translational Medicine, Sant’Andrea Hospital of Rome, “Sapienza” University of Rome, 00189 Rome, Italy; tipaldi.andrea@gmail.com; 7 Department of Medical and Surgical Sciences (DIMEC), University of Bologna, 40136 Bologna, Italy

**Keywords:** pulmonary embolism, catheter-directed therapy, mechanical thrombectomy, PERT, right ventricular dysfunction, risk stratification, CT pulmonary angiography

## Abstract

Acute pulmonary embolism (PE) is increasingly managed as a dynamic risk continuum in which imaging findings guide therapeutic escalation rather than merely confirm diagnosis. The principal challenge still remains normotensive patients with intermediate–high-risk features, where early right ventricular (RV) dysfunction may precede overt hemodynamic collapse. New trends focus on a trajectory-based model by integrating clinical, laboratory, and standardized imaging parameters into severity categorization. This review critically examines how imaging-derived markers influence risk stratification, escalation timing, and endovascular decision pathways in contemporary PE management. A structured narrative review was conducted focusing on the literature published between January 2020 and January 2026. PubMed/MEDLINE, Scopus, and Web of Science were searched for studies addressing imaging-based risk assessment, catheter-based reperfusion strategies, randomized trials, prospective registries, and guideline documents. Contemporary data consistently demonstrate that catheter directed therapies (CDTs) lead to rapid improvement in RV imaging surrogates and hemodynamic parameters. However, short-term mortality differences are uncommon in predominantly normotensive cohorts. Clinically meaningful signals instead emerge in the reduction in early clinical deterioration, the need for rescue escalation, bleeding optimization, and healthcare resource utilization. Imaging, as standardized reporting of RV strain on computed tomography pulmonary angiography and echocardiography, should be further embedded into escalation algorithms. In modern PE care, imaging functions as a trigger for escalation within multidisciplinary pathways rather than as a passive prognostic marker. CDTs should be interpreted as tools for trajectory modulation in selected intermediate-risk patients rather than mortality-reduction strategies. Future research should integrate imaging phenotyping, dynamic reassessment models, and organizational variables to refine patient selection and optimize outcome-relevant endpoints.

## 1. Introduction

Acute pulmonary embolism (PE) remains a time-sensitive condition in which early adverse outcomes are primarily mediated by acute right ventricular (RV) failure triggered by abrupt pulmonary vascular obstruction and vasoconstriction. Contemporary management, however, has progressively moved beyond a binary diagnostic paradigm toward a risk continuum in which imaging contributes to clinical decision-making beyond diagnosis. The 2019 European Society of Cardiology (ESC) guidelines operationalized this continuum by combining hemodynamic status, clinical risk scores (PESI/sPESI), and objective markers of RV dysfunction and myocardial injury derived from computed tomography pulmonary angiography (CTPA)/echocardiography and biomarkers [1,2,3].

Within the ESC framework, reperfusion is mandatory in high-risk PE, whereas anticoagulation alone remains standard for most intermediate-risk cases. The persistent clinical challenge lies in the intermediate–high-risk subgroup: patients who are normotensive at presentation yet biologically unstable, with a non-trivial probability of early deterioration. In this setting, imaging findings are frequently used as operational triggers including RV/LV ratio, RV strain patterns, thrombus distribution, and clot centrality, because they provide a time window in which RV pressure overload may still be reversible (Table 1). Importantly, the recently released 2026 AHA/ACC multisociety guideline reinforces this approach by recommending that, when CTPA is performed, parameters of RV strain should be reported explicitly, including a numerical RV/LV ratio, and by detailing a comprehensive echocardiographic assessment of RV dysfunction/strain based on local expertise (Table 2) [4,5,6,7].

Parallel to this imaging-enabled risk refinement, endovascular reperfusion strategies, including catheter-directed therapies (CDTs), with or without ultrasound-assisted thrombolysis (USAT) and mechanical thrombectomy (MT), have expanded rapidly as alternatives to systemic thrombolysis, aiming to reduce RV afterload through targeted thrombus debulking and/or localized fibrinolysis while limiting major bleeding. In the same period, Pulmonary Embolism Response Teams (PERTs) have evolved from ad hoc consultation models into governance structures that integrate clinical and organizational factors into escalation decisions. Randomized trials and large registries published in the last 5–6 years have therefore begun to shift the evidentiary focus away from physiology alone toward clinically meaningful outcomes, deterioration, rescue escalation, bleeding, intensive care utilization, and length of stay, where differences are more likely to emerge than in short-term mortality for predominantly normotensive cohorts [4,8,9].

Despite this rapid adoption, the role of CDTs and MT within guideline-directed management remains incompletely defined, especially in intermediate–high-risk PE, because early improvement in RV imaging surrogates does not automatically translate into patient-centered benefit. Moreover, organizational variables introduced by PERT-enabled pathways may influence outcomes as much as device-specific effects, complicating interpretation across heterogeneous study designs and endpoints. Against this background, radiology, and particularly interventional radiology (IR), has transitioned from a diagnostic support function to an active determinant of risk stratification, escalation timing, and image-guided reperfusion strategy [10,11,12,13].

In this context, a key unresolved question in contemporary PE management is whether imaging-derived parameters can meaningfully guide therapeutic escalation in normotensive patients with intermediate-risk features, and how such imaging signals should be interpreted within multidisciplinary decision pathways. Accordingly, this structured narrative review critically examines contemporary evidence (2020–2026) through a radiology-centered lens, focusing on: (i) imaging-derived markers used to guide patient selection and escalation timing; (ii) feasibility and rationale of endovascular reperfusion across the ESC risk continuum; (iii) how PERT governance operationalizes imaging-to-decision workflows; and (iv) how contemporary trials and registries should be interpreted when mortality is an infrequent endpoint and trajectory-related outcomes beyond mortality increasingly dominate clinical relevance.

**Table 1:** Evolution of risk classification frameworks in acute pulmonary embolism: from ESC 2019 to AHA/ACC 2026 and implications for imaging-guided escalation.

Domain	ESC 2019 Model	AHA/ACC 2026 Model	Implications for Imaging-Guided Management
Conceptual approach	Static mortality-based stratification	Dynamic vulnerability and trajectory-based model	Imaging contributed to early identification of instability
Intermediate-risk definition	Broad, heterogeneous category	Sub phenotype based classification	Improved patient selection for CDT/MT
Role of Imaging	Prognostic marker (RV dysfunction)	Integrated component of severity assessment	Imaging becomes actionable in escalation decisions
Therapeutic strategy	Step-up rescue approach	Pre-emptive trajectory modulation	Supports earlier intervention in selected patients
Organization Model	Variable non-standardized	Multidisciplinary pathway (PERT-based)	Facilitates structured escalation decisions

**Table 2:** Conceptual transition from static risk stratification to trajectory-based severity assessment in acute pulmonary embolism.

Conceptual Domain	ESC 2019 Framework	AHA/ACC 2026 Framework	Clinical Impact
**Philosophy**	Mortality risk prediction	Dynamic clinical severity assessment	Shift toward trajectory-based care
**Risk categories**	Discrete data (low → high risk)	Continuous severity spectrum (A–E)	Reflects real-word heterogeneity
**Role of instability**	Binary threshold (shock vs. no shock)	Extreme of a severity continuum	Enables earlier recognition of deterioration
**Imaging**	Risk stratification tool	Decision-support biomarker	Guides escalation pathways
**Monitoring**	Reactive (intermediate–high-risk)	Proactive across severity spectrum	Enables early trajectory detection
**Reperfusion strategy**	Rescue therapy	Integrated escalation option	Supports individualized intervention

## 2. Materials and Methods

*Design and Scope of the Review.* This study was conducted as a structured narrative review aimed at providing a clinically actionable, imaging-centered interpretation of contemporary evidence on acute PE management. The review was not designed as a systematic review or meta-analysis; rather, its purpose was to contextualize emerging trial data, registry findings, and guideline recommendations within real-world decision-making processes involving diagnostic and interventional imaging. The analytical focus was therefore placed on how imaging findings influence risk reassessment, escalation timing, and selection of endovascular reperfusion strategies.

*Literature Search Strategy.* A focused literature search was performed using PubMed/MEDLINE as the primary database, complemented by Scopus and Web of Science to ensure adequate coverage of radiology, interventional, cardiovascular, and critical care publications. The search timeframe extended from 1 January 2020 to 31 January 2026, selected to reflect the contemporary clinical era following the implementation of the 2019 ESC risk-stratification model and the subsequent expansion of CDTs. To contextualize current evidence, selected landmark studies published before 2020 were also considered, when necessary, particularly when they provided foundational data on CDTs. Search terms combined controlled vocabulary and free-text keywords related to PE and imaging-guided management, including: pulmonary embolism, catheter-directed therapy, catheter-directed thrombolysis, ultrasound-assisted thrombolysis, mechanical thrombectomy, aspiration thrombectomy, risk stratification, right ventricular dysfunction, CT pulmonary angiography, and Pulmonary Embolism Response Team (PERT). Reference lists of major guidelines, consensus statements, and pivotal clinical studies were manually screened to identify additional relevant publications.

*Study Selection and Evidence Prioritization.* Given the interpretative objective of the review, studies were selected based on their ability to inform clinically meaningful domains rather than according to predefined quantitative inclusion or exclusion criteria. Priority was given to randomized controlled trials, prospective multicenter registries, guideline and consensus documents, and high-quality observational studies addressing outcomes relevant to therapeutic escalation and clinical trajectory. Overall, approximately 70 publications were considered most relevant to the narrative synthesis, including randomized trials evaluating CDTs, prospective registries of endovascular reperfusion strategies, and contemporary guideline documents. Smaller descriptive series were considered when they provided specific insights into imaging assessment, procedural feasibility, or workflow integration. Particular attention was paid to ensuring balanced representation of the available literature, including studies evaluating both CDTs and MT, as well as investigations addressing imaging-based risk stratification and multidisciplinary management models. To improve transparency, the selection process was guided by predefined thematic domains, including imaging-based risk stratification, RV dysfunction assessment, escalation criteria, and CDT strategies. Studies were screened for relevance to these domains and prioritized based on their contribution to clinically applicable decision-making frameworks.

*Narrative Synthesis Approach.* Given the heterogeneity in study design, patient populations, and endpoint definitions, findings were synthesized qualitatively through comparative critical analysis rather than statistical pooling. The synthesis focused on identifying patterns of evidence relevant to clinical decision-making, including relationships between imaging findings, risk reassessment, and therapeutic strategies. Emphasis was placed on reconciling differences between surrogate physiological measures and clinically meaningful outcomes, as well as on understanding how study design, patient selection, and multidisciplinary care models may influence the interpretation and applicability of published results.

## 3. Limits of Static Risk Stratification

Risk stratification models have been central to modern PE management, providing a structured framework to estimate early mortality and guide the intensity of initial therapy. The 2019 ESC classification integrates hemodynamic status, clinical severity scores, biomarkers, and imaging evidence of RV dysfunction to categorize patients into low-, intermediate-, and high-risk groups. This model has significantly improved consistency in therapeutic decision-making and remains the cornerstone of contemporary guideline-directed care [1].

However, these categories represent a static approximation of a condition that is intrinsically dynamic. In clinical practice, deterioration rarely occurs as an abrupt transition between predefined classes, but rather develops progressively through increasing RV pressure overload, ventricular–arterial uncoupling, and eventual compensatory failure. As a result, patients classified as intermediate risk at presentation may follow markedly different clinical courses, ranging from rapid stabilization under anticoagulation to delayed hemodynamic compromise despite initially reassuring systemic parameters [14,15].

This limitation is particularly relevant in intermediate high-risk PE, where the choice between anticoagulation alone and escalation to reperfusion therapies cannot be determined solely by baseline classification. In this setting, ongoing clinical and imaging evaluation becomes essential to identify early signs of unfavorable evolution. A static framework therefore requires a framework capable of capturing temporal changes in RV function, thrombus burden, and cardiopulmonary [16].

Recent multisociety recommendations acknowledge this need by emphasizing the importance of structured evaluation of RV dysfunction within ongoing clinical assessment rather than relying on single-point categorization. This perspective reflects a shift toward anticipating disease evolution under treatment rather than assigning a fixed risk label [7,17].

### 3.1. Imaging as a Tool for Dynamic Reassessment

In acute PE, imaging has traditionally been interpreted as a diagnostic and prognostic tool. In contemporary practice, however, its role extends beyond initial assessment to support longitudinal evaluation of RV adaptation under persistent afterload stress. Rather than acting as a static marker of severity, imaging provides objective parameters that, when integrated over time, contribute to clinical regarding monitoring, escalation, and treatment response.

In this context, imaging findings may serve three interconnected functions. First, they provide baseline prognostic information reflecting physiological severity, such as the RV/LV ratio on CTPA. Second, they contribute to escalation decisions when interpreted alongside evolving clinical and laboratory parameters. Third, they allow assessment of treatment response following anticoagulation or endovascular reperfusion. The relationship between imaging phenotypes and potential escalation strategies is summarized in Table 3. Recognizing these complementary roles helps avoid misinterpreting imaging parameters as isolated treatment triggers rather than as components of an integrated clinical framework [16,17].

CTPA remains the primary diagnostic modality and enables immediate assessment of RV strain through parameters such as RV/LV diameter ratio, septal bowing, contrast reflux into the inferior vena cava, and clot distribution. These findings reflect ventricular/arterial interaction at a given time point and gain clinical relevance when interpreted in combination with temporal evolution and clinical context. In particular, they may help identify patients whose apparent hemodynamic stability conceals early signs of decompensation [18].

Echocardiography complements CTPA by providing real-time evaluation of RV function, including contractility patterns, pressure overload, tricuspid annular plane systolic excursion (TAPSE), and strain-derived indices when available. Additional parameters such as the right ventricular Tei index (myocardial performance index), which provides a more global assessment of RV function and may be less influenced by geometric assumptions and probe angulation, can offer complementary information. In this setting, its value lies less in confirming the diagnosis and more in detecting evolving dysfunction despite anticoagulation, thereby refining the timing of potential escalation [19].

Recent multisociety recommendations emphasize standardized reporting of RV dysfunction parameters, particularly numerical RV/LV ratio assessment on CTPA. This structured approach facilitates reproducible communication within multidisciplinary teams and reduces variability in clinical interpretation, supporting a shared decision-making process within PERT [20,21].

Importantly, imaging findings should not be interpreted in isolation. An elevated RV/LV ratio in a clinically stable patient may justify close monitoring, whereas the same finding in the presence of worsening biomarkers, persistent tachycardia, or deteriorating gas exchange may indicate impending instability. The clinical interpretation of imaging findings requires consideration of their evolution over time [7,22].

Within this framework, imaging supports a shift from categorical classification toward a longitudinal, context-driven approach in which repeated assessment informs whether anticoagulation alone is sufficient or early image-guided reperfusion should be considered.

**Table 3:** Imaging phenotypes and potential escalation implications in acute pulmonary embolism.

Imaging Phenotype	Clinical Interpretation	Associated Risk Profile	Suggested Management Approach
RV/**LV ratio** ≥ 1 with septal bowing	Significant RV pressure overload	Intermediate high-risk	Close monitoring ± early PERT discussion
Progressive RV dysfunction (serial echo)	Worsening ventricular failure	High risk of deterioration	Consider early escalation (CDT/MT)
Central clot burden without RV dysfunction	Anatomical severity without hemodynamic compromise	Stage C–D	Anticoagulation with surveillance
RV dysfunction + biomarker elevation	Hemodynamic vulnerability	Stage D–E	Early multidisciplinary evaluation
Persistent RV strain despite anticoagulation	Failure of initial therapy	Evolving instability	Consider escalation strategy

### 3.2. The Escalation Window in Intermediate-Risk Pulmonary Embolism

The main challenge in contemporary PE management does not lie in identifying high-risk patients requiring immediate reperfusion, nor in managing low-risk cases suitable for anticoagulation and early discharge. Rather, it concerns the intermediate high-risk subgroup, in whom hemodynamic stability coexists with objective evidence of RV dysfunction and myocardial injury. In these patients, decisions regarding escalation beyond anticoagulation depend less on baseline classification and more on the evolution of clinical and imaging parameters over time [16,17,18,19,20,21,22,23].

The concept of an “escalation window” reflects the recognition that RV failure in acute PE develops progressively. Compensatory mechanisms may initially preserve systemic blood pressure despite significant pressure overload, but sustained ventricular–arterial uncoupling can eventually lead to abrupt decompensation. The therapeutic objective is therefore not only to treat established instability, but also to identify a potentially reversible phase in which early intervention may prevent further deterioration. This perspective is increasingly reflected in recent multisociety recommendations, which emphasize integrated reassessment of clinical status, biomarkers, and standardized imaging parameters rather than reliance on single-point risk categorization [1,24].

Imaging plays a key role in identifying this phase. Progressive RV dilation, worsening septal bowing, declining echocardiographic indices of contractility, or persistent RV strain in association with rising biomarkers may indicate a reduced compensatory reserve. In this context, emerging concepts such as normotensive shock, defined by preserved systemic blood pressure in the presence of impaired cardiac output and/or markers of systemic hypoperfusion, further highlight the limitations of binary hemodynamic classification. Composite risk models, including the Composite PE Shock score, have been proposed to identify patients at higher risk of deterioration despite apparent hemodynamic stability. These approaches reinforce the importance of integrating imaging findings with physiological and biochemical indicators to refine escalation decisions. Within this framework, individual parameters acquire clinical significance primarily when interpreted in context. An isolated abnormal RV/LV ratio does not mandate intervention, whereas persistent or worsening dysfunction despite anticoagulation may support consideration of escalation. In parallel, point-of-care ultrasonography may represent a practical adjunct for bedside reassessment, allowing rapid and repeatable evaluation of RV parameters. While less comprehensive than formal echocardiography, it may support integration of imaging findings into dynamic clinical assessment [7,15,25].

In this framework, escalation is not triggered by risk category alone but by the convergence of multiple signals, including imaging findings, biomarker trends, clinical stability, oxygenation, and local expertise. A structured reassessment within the first 24–72 h should integrate imaging (RV/LV ratio or echocardiographic dysfunction), biomarker trends, and clinical parameters such as oxygenation and heart rate to identify patients with early signs of unfavorable evolution who may benefit from escalation. In patients with intermediate-risk PE, the combination of persistent RV dysfunction on imaging, rising cardiac biomarkers, and signs of clinical instability such as worsening oxygenation or sustained tachycardia may support consideration of escalation beyond anticoagulation. Conversely, isolated imaging abnormalities in clinically stable patients may justify continued monitoring and reassessment rather than immediate intervention [26].

This approach also helps explain the apparent discrepancy observed in contemporary trials, where improvements in RV imaging parameters are not consistently associated with mortality reduction. In normotensive populations with relatively low baseline mortality, the benefit of intervention is more likely to be reflected in reduced clinical deterioration, lower need for rescue therapies, shorter intensive care stay, and potentially improved safety profiles compared with systemic thrombolysis. In this context, the timing of escalation, rather than the specific device used, may represent a critical determinant of clinical benefit [27].

Recognizing and operationalizing the escalation window requires structured reassessment strategies and effective communication within multidisciplinary teams. Without systematic integration of imaging into ongoing clinical evaluation, opportunities for timely intervention may be missed.

### 3.3. Endovascular Therapies Within a Trajectory-Based Model

Within this clinical context, CDTs are defined not by the technologies employed but by the clinical objective they aim to achieve, namely reduction in RV afterload during a phase of potentially reversible dysfunction. CDTs, including local thrombolytic infusion with or without USAT, and MT represent complementary strategies that target pulmonary vascular obstruction, although the level and quality of supporting evidence differ across available studies [28,29,30,31,32,33].

The rationale for these interventions lies in achieving rapid reduction in pulmonary vascular resistance and RV wall stress while limiting systemic exposure to thrombolytic agents. In intermediate-risk PE, their role is not primarily as rescue therapy, but as a potential means to prevent clinical deterioration in selected patients showing signs of insufficient response to anticoagulation [12,13].

From this perspective, the distinction between pharmacological and mechanical approaches is secondary to patient selection, timing of intervention, and bleeding risk. Local fibrinolysis may be appropriate when a gradual reduction in thrombus burden is acceptable and hemorrhagic risk is manageable, whereas MT offers the possibility of immediate hemodynamic improvement without thrombolytic exposure. Both strategies ultimately aim to reduce RV pressure overload and should be considered within the context of clinical presentation, institutional expertise, and individual risk profile [10,11,34,35,36].

Importantly, the effectiveness of these therapies should not be assessed solely in terms of technical success or angiographic thrombus reduction. Their clinical value lies in their potential to reduce the risk of hemodynamic deterioration, limit the need for rescue systemic thrombolysis, shorten intensive care utilization, and support earlier cardiopulmonary recovery. These outcomes may be particularly relevant in normotensive patients, in whom mortality is relatively low and less sensitive to treatment-related differences [1,6,7].

Accordingly, CDTs should be viewed as a component of guideline-directed management, representing a targeted escalation strategy in patients with evolving clinical and imaging findings suggesting that anticoagulation alone may be insufficient.

### 3.4. Reinterpreting Contemporary Evidence Through a Trajectory Lens

Although the formal search focused on 2020–2026, selected landmark earlier trials were also considered when necessary to contextualize current evidence; an expanding body of studies has evaluated CDTs and MT in acute PE. Randomized and prospective investigations, including trials such as ULTIMA and OPTALYSE-PE as well as large prospective registries such as SEATTLE II, FLARE, and EXTRACT-PE, have consistently demonstrated rapid improvements in surrogate markers of right ventricular dysfunction, including reductions in RV/LV ratio, pulmonary artery pressures, and indices of myocardial strain [13,22,30,31,33,37].

Despite these reproducible physiological effects, randomized trials and prospective registries have not shown a consistent reduction in short-term mortality, particularly in predominantly normotensive populations [38,39,40,41].

This apparent discrepancy has often been interpreted as evidence of limited clinical benefit. However, such a conclusion may reflect a mismatch between the outcomes measured and the therapeutic objective of intervention. In intermediate-risk PE, baseline mortality under contemporary anticoagulation is already low, rendering mortality an insensitive endpoint for detecting the value of therapies intended to prevent deterioration rather than to rescue established shock [14,26].

When interpreted in relation to disease progression, the findings of these studies appear more coherent. In this perspective, improvements in imaging surrogates reported across multiple studies may be interpreted as indicators of early trajectory modification rather than direct determinants of survival benefit. Early RV unloading, reduction in clot burden, and rapid hemodynamic stabilization may translate into differences in clinical course that are not captured by mortality alone, including avoidance of rescue systemic thrombolysis, reduced escalation to vasopressor or ventilatory support, shorter intensive care stay, and accelerated functional recovery. These outcomes, although variably defined across studies, align more closely with the concept of trajectory modification introduced earlier [10,11,12,13,34].

Heterogeneity in trial design further complicates interpretation. It is also important to recognize that CDTs and MT have been evaluated through different evidentiary pathways. Randomized trials have predominantly investigated thrombolysis-based strategies, whereas most data on MT derive from prospective single-arm studies and registries. This methodological heterogeneity limits direct comparison between technologies and complicates the extrapolation of findings across different intervention types and patient populations. As a result, treatment effects observed in selected cohorts may not be generalizable to the broader population of patients with intermediate-risk PE, and differences in timing, patient selection, and clinical context must be carefully considered when interpreting available evidence. These considerations further support a cautious interpretation of current data and highlight the importance of individualized, context-dependent decision-making. Variations in inclusion criteria, timing of intervention, imaging thresholds used to define RV dysfunction, and institutional expertise influence which patients are treated and when, thereby affecting the magnitude of observable benefit. In many cases, interventions may have been applied either too early, before unfavorable evolution was evident, or too late, after significant RV injury had already occurred, blurring the measurable impact [21,24]. Key clinical studies evaluating CDTs and MT are summarized in Table 4.

Ongoing randomized trials, such as STRIKE-PE, are expected to provide additional data on early clinical and hemodynamic outcomes and may contribute to refining patient selection and timing of intervention. From this standpoint, contemporary evidence should not be viewed as testing whether CDT “works” in a binary sense, but rather as exploring how patient selection, timing, and multidisciplinary integration determine its effectiveness. The challenge for future studies is therefore less about comparing devices and more about refining imaging-driven selection strategies and defining clinically meaningful endpoints capable of capturing prevention of deterioration along the PE trajectory [6,36].

**Table 4:** Key clinical studies evaluating CDTs acute PE.

Study	Design	Population	Intervention	Primary Endpoint	Key Findings	Clinical Interpretation	Main Limitations
**ultima**	Randomized controlled trial	Intermediate high-risk PE	USAT vs. anticoagulation	RV/LV ratio reduction at 24 h	Significant improvement in RV function with CDTs	Demonstrates physiological efficacy of CDTs	Small sample size, surrogate endpoint
**optalyse-pe**	Randomized controlled trial (dose/duration)	Intermediate high-risk PE	USAT (various regimens)	RV/LV ratio reduction	Shorter and lower-dose regimens effective	Supports feasibility and optimization of thrombolysis strategies	No clinical outcome endpoints
**seattle II**	Prospective multicenter, single-arm	Massive and submassive PE	USAT	RV/LV ratio reduction and pulmonary artery pressure	Marked RV improvement and PA pressure reduction	Confirms reproducible hemodynamic benefit of CDT	No control group
**flare**	Prospective multicenter	Intermediate high-risk PE	MT	RV/LV ratio reduction at 48 h	Rapid RV unloading without thrombolytics	Supports MT as non-lytic alternative	Single-arm design
**extract-pe**	Prospective multicenter	Intermediate high-risk PE	MT	RV/LV ratio reduction at 48 h	Significant RV improvement with low bleeding risk	Reinforces role of MT in selected patients	No comparator arm
**flash ** **registry**	Prospective real-word registry	Intermediate high-risk PE	MT	Safety and hemodynamic outcomes	Rapid clinical and hemodynamic improvement	Reflects real-world applicability of MT	Observational design
**peerless**	Randomized controlled trial	Intermediate high-risk PE	MT vs. Catheter directed thrombolysis	Composite clinical endpoint	MT showed favorable outcomes in selected patients	Suggests potential role of MT beyond surrogate improvement	Heterogeneous endpoints
**storm-pe**	Randomized controlled trial	Intermediate high-risk PE	MT vs. anticoagulation	Composite outcome (clinical deterioration)	Early signals of benefit in preventing deterioration	Aligns with trajectory-based escalation concept	Ongoing interpretation and limited follow-up

### 3.5. PERT as the Operational Interface of Trajectory-Based Care

Trajectory-based management in acute PE requires more than refined risk categorization and advanced imaging; it demands a coordinated system capable of translating evolving clinical and radiological signals into timely therapeutic decisions. PERT have emerged as the structural interface through which this translation occurs [4,9,42].

Originally conceived to streamline consultation for complex PE cases, PERT models have progressively evolved into multidisciplinary platforms integrating cardiology, pulmonology, emergency medicine, intensive care, hematology, and interventional radiology. Within a trajectory-oriented framework, their principal value lies not simply in expediting reperfusion therapy, but in enabling structured evaluation and shared interpretation of clinical data [9,43].

Imaging occupies a central position within this process. Standardized reporting of RV strain parameters on CTPA, serial echocardiographic evaluation, and integration of biomarker trends provide a common evidentiary substrate for multidisciplinary discussion. The decision to escalate from anticoagulation to CDTs is therefore not a unilateral procedural choice, but the outcome of collective interpretation of trajectory signals. This approach reduces operator-dependent variability and aligns escalation decisions with shared multidisciplinary thresholds rather than individual procedural bias [6,18,19].

Importantly, PERT implementation may also reduce variability in escalation thresholds across institutions. By formalizing communication pathways and establishing predefined criteria for monitoring and reassessment, these teams mitigate the risk of both premature intervention and delayed rescue. In this sense, PERT structures operationalize the escalation window concept described earlier, transforming it from theoretical construct to actionable workflow. Within this organizational framework, the role of PERT extends beyond in-hospital decision-making to include coordination of interhospital transfer in selected patients. In institutions lacking advanced interventional capabilities or dedicated expertise, early identification of patients with unfavorable trajectories may prompt transfer to referral centers equipped with endovascular treatment options. This hub-and-spoke approach reflects the trajectory-based model, in which escalation is not only procedural but also organizational, requiring timely recognition of deterioration risk and appropriate allocation of resources. Such strategies may be particularly relevant in preventing delayed escalation and ensuring access to advanced therapies in appropriate candidates [43,44].

Beyond acute decision-making, PERT models facilitate longitudinal follow-up and post-discharge assessment, ensuring that early trajectory modification translates into sustained cardiopulmonary recovery. As such, they represent not merely a consultative service but an organizational embodiment of trajectory-based care. At the same time, important sources of variability remain. The structure, activation criteria, and operational workflows of PERT programs differ substantially across institutions, reflecting differences in local expertise, available interventional capabilities, and institutional culture. In many settings, escalation protocols are not fully standardized, and the decision to activate a PERT may itself introduce selection bias by focusing attention on more complex or severe cases. These organizational factors should therefore be considered when interpreting observational data on endovascular therapies and multidisciplinary PE management. Accordingly, any apparent association between PERT implementation and improved clinical outcomes should be interpreted with caution, as current evidence does not establish a direct causal relationship [45,46].

Rather than introducing a new paradigm, this framework aims to provide a structured interpretation of already established clinical, imaging, and physiological principles. Its contribution lies in integrating these elements into a more explicit and operational approach to patient monitoring and treatment escalation in acute PE.

## 4. Discussion

Contemporary management of acute PE is evolving beyond static risk stratification toward a more integrated approach that incorporates repeated clinical, imaging, and biomarker assessment. While traditional prognostic models remain essential for initial evaluation, they may incompletely reflect the dynamic nature of cardiopulmonary impairment in acute PE [1,14,16,17]. The proposed framework does not aim to replace existing guideline-based risk stratification models, but rather to provide a structured, imaging-centered interpretation of these principles, with the goal of supporting real-world clinical decision-making, particularly in intermediate-risk patients. Importantly, its contribution lies not in redefining risk categories, but to guide clinical decision-making over time. In this sense, it should be viewed as a pragmatic operationalization of existing recommendations rather than a novel classification system.

In this context, combining imaging findings with clinical and laboratory parameters allows a more refined assessment of disease evolution during anticoagulation. Patients initially classified within the same risk category may follow different clinical courses, ranging from stable recovery to progressive RV dysfunction and hemodynamic deterioration. The clinical challenge therefore lies in identifying early signs of unfavorable evolution among apparently stable patients [15,21,24].

**Figure 1:**
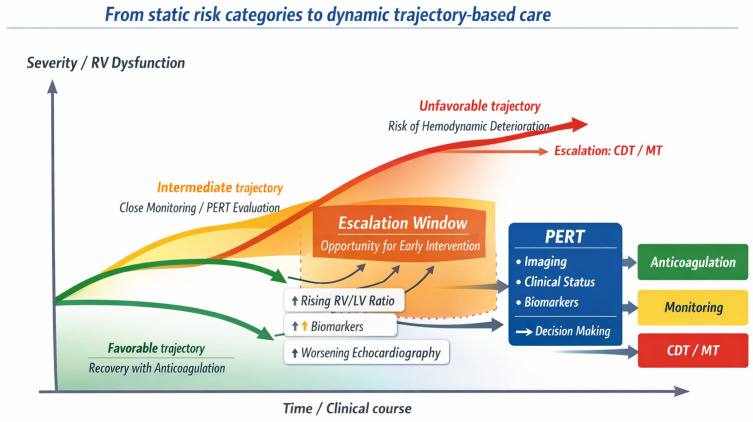
Trajectory-based model of acute PE management. Acute PE should be interpreted as a dynamic clinical continuum rather than a static risk category. Patients may evolve along different trajectories over time, ranging from favorable recovery under anticoagulation to progressive RV dysfunction and hemodynamic deterioration. Imaging findings, including RV/LV ratio on CTPA and echocardiographic parameters, together with biomarkers and clinical variables, contribute to continuous risk reassessment. An intermediate “escalation window” may be identified in selected patients with evolving RV dysfunction, where early CDts may prevent clinical deterioration. Multidisciplinary evaluation through PERT integrates these signals into individualized decision-making pathways.

From a practical perspective, the trajectory-based framework translates into a structured approach to sequential reassessment during anticoagulation. Initial risk stratification should be complemented by early re-evaluation of RV function, biomarker trends, and clinical status within the first 24–72 h. In patients showing stable or improving parameters, conservative management may be continued. Conversely, the combination of persistent RV dysfunction, worsening imaging findings, or rising biomarkers despite anticoagulation may identify a subgroup at increased risk of clinical deterioration, in whom escalation to endovascular therapies may be considered. In this setting, the framework does not replace guideline-based recommendations, but provides a clinically actionable lens to interpret dynamic changes over time and to support individualized escalation decisions (Figure 1).

This perspective also helps interpret the results of contemporary trials evaluating endovascular reperfusion strategies. Several studies have demonstrated rapid improvements in RV function following CDTs, yet these physiological effects have not consistently translated into mortality reduction, particularly in normotensive populations. It should be emphasized that improvements in imaging-derived parameters, including RV/LV ratio and other markers of RV dysfunction, represent physiological indicators of treatment response and are not validated surrogate endpoints for clinically meaningful outcomes such as mortality or long-term functional recovery. Therefore, their clinical interpretation should remain cautious and always integrated with patient-centered outcomes and clinical context. In such settings, the clinical benefit of intervention may be better reflected by reduced rates of hemodynamic deterioration, decreased need for rescue therapies, shorter intensive care stay, and improved early functional recovery rather than by differences in survival alone [38,39,40,47,48].

Recent guideline updates further support a move toward integrated assessment of disease severity. The increasing emphasis on standardized evaluation of RV dysfunction and its incorporation into ongoing clinical decision-making highlights the growing role of imaging beyond initial diagnosis [1,6,36,49,50].

From a practical standpoint, multidisciplinary models such as PERTs provide a structured environment for coordinated, real-time decision-making within a multidisciplinary governance model. These systems facilitate shared decision-making and may help reduce variability in patient selection and timing of escalation [4,43,44].

Despite these advances, several limitations remain. First, there is limited consensus regarding imaging thresholds that should prompt escalation to endovascular therapy. Although the RV/LV diameter ratio on CTPA is widely used, cutoff values vary across studies, typically ranging from 0.9 to ≥1.0. Echocardiographic parameters such as TAPSE, RV strain, and qualitative assessment of RV dilation are also commonly applied, but their reproducibility and thresholds may differ depending on operator expertise and institutional practice [8,18,19,51]. As a result, no universally validated imaging criteria currently exist to mandate escalation to CDTs.

Second, available randomized trials are heterogeneous in terms of study design, inclusion criteria, and endpoints, limiting direct comparison between different therapeutic approaches. In addition, different CDTs are supported by heterogeneous levels of evidence. Randomized controlled trials are available for thrombolysis-based approaches, whereas most data on MT derive from prospective single-arm registries. These differences should be considered when interpreting the apparent consistency of physiological and clinical outcomes across studies [10,11,29,52]. Third, most studies focus on short-term physiological outcomes, whereas long-term clinical implications, including functional recovery and prevention of chronic thromboembolic disease, remain insufficiently defined. In this context, post-PE syndrome represents an important and often under-recognized dimension of disease burden. A substantial proportion of patients experience persistent dyspnea, exercise limitation, and impaired quality of life despite adequate anticoagulation, even in the absence of overt chronic thromboembolic pulmonary hypertension. These patient-centered outcomes are increasingly recognized as clinically relevant endpoints. Early reduction in RV strain and more rapid hemodynamic recovery following CDTs may potentially contribute to improved medium-term functional status, although robust evidence remains limited. Ongoing studies incorporating patient-reported outcomes, such as PE Quality of Life (QoL), may help clarify the impact of these interventions beyond traditional clinical endpoints [45,46].

Future research should therefore focus on imaging-driven patient phenotyping and on the identification of clinically meaningful endpoints beyond mortality. In particular, outcomes such as prevention of clinical deterioration, reduction in intensive care utilization, and accelerated recovery may better capture the impact of endovascular therapies in intermediate-risk populations [53,54].

In summary, acute PE management is increasingly oriented toward an approach in which imaging-guided reassessment, multidisciplinary coordination, and timely escalation decisions contribute to individualized care. Within this framework, CDTs should be considered as part of a broader therapeutic strategy rather than as isolated procedural interventions [1,36].

This review has several limitations. As a structured narrative review, it reflects an interpretative synthesis rather than a formal systematic evaluation of all available evidence. Although priority was given to randomized trials, major registries, and guideline documents, selection bias cannot be excluded. Furthermore, heterogeneity across studies in terms of patient populations, definitions, and outcomes limits direct comparison. These factors should be considered when interpreting the framework proposed in this review.

## 5. Conclusions

The management of acute PE is progressively moving beyond static risk stratification toward a dynamic model centered on clinical trajectory and physiological vulnerability. Within this evolving framework, imaging assumes a pivotal role not only in diagnosis but also in guiding reassessment, identifying early signals of deterioration, and informing escalation decisions.

Endovascular reperfusion strategies should therefore be interpreted as tools for trajectory modulation rather than as interventions primarily aimed at reducing mortality in normotensive populations. Their clinical value lies in stabilizing RV function, preventing hemodynamic deterioration, and supporting recovery when anticoagulation alone may be insufficient.

The integration of imaging findings, biomarker trends, and multidisciplinary evaluation, often operationalized through PERT, enables a more individualized approach to patient management. Future research should focus on refining imaging-driven phenotyping, standardizing escalation thresholds, and defining outcome measures that better capture prevention of clinical deterioration and functional recovery.

## Data Availability

No new data were created or analyzed in this study.
